# The Half-Yearly Compendium of Medical Science

**Published:** 1867-11

**Authors:** 


### THE HALF-YEARLY COMPENDIUM OF MEDICAL SCIENCE,

Being a Synopsis of Practical Medicine, Surgery, and Medical Literature.

The first part of this work will be issued from the office of the *Medical and Surgical Reporter* on the first of January, 1868. It will comprise about 300 pages royal octavo size, and will contain a well-prepared synopsis of the articles in the medical periodicals and monographs, and a general review of the medical literature of the preceding six months, both of this country and Europe.

In the preparation of this work we will be aided by many well-known writers, among whom are Drs. L. Elsburg, Samuel R. Percy, R. E. Van Gieson, F. D. Weisse, C. F. J. Lehlback, S. W. Gross, George H. Napheys, W. M. Turner, A. Paul Turner, and others.

The work will be systematically arranged under the following heads—subject to such modifications as time and experience may suggest—and supplied with a copious Index of subjects and authors.

*Terms of Subscription.*—Subscriptions will now be received at the following rates:

| | | |
|---|---|---|
| Compendium, per annum, two numbers, | - - - - - - | $3 00 |
| " Single number, | - - - - - - - - | 2 00 |
| " and Medical and Surgical Reporter, per annum, | - | 7 00 |

S. W. BUTLER, M. D. }

D. G. BRINTON, M. D. } Editors.

115 South Seventh St., Philadelphia.

